# A Time-Based and Clinical Status Stratified Protocol for Major Bile Duct Injury After Cholecystectomy: Retrospective, Single-Center Outcomes From a Resource-Limited Setting

**DOI:** 10.7759/cureus.102086

**Published:** 2026-01-22

**Authors:** Ahmed Ateik, Saif A Ghabisha, Lamia Abdulmughni, Fares Awn

**Affiliations:** 1 Department of General Surgery, Faculty of Medicine, 21 September University for Medical and Applied Sciences, Sana’a, YEM; 2 Department of General Surgery, Ibb University, Ibb, YEM; 3 Department of Surgery, University of Science and Technology Hospital, Sana'a, YEM

**Keywords:** bile duct injury, cholecystectomy, hepaticojejunostomy, management protocol, resource-limited settings, strasberg classification, surgical outcomes

## Abstract

Background

The management of major iatrogenic bile duct injury (BDI) (Strasberg types D and E) is critically influenced by the timing of repair and patient physiology, posing a significant clinical challenge in resource-constrained environments. This study aimed to determine the optimal timing for surgical intervention, identify predictors of repair failure, and evaluate long-term anastomotic patency and postoperative complications in patients undergoing Roux-en-Y hepaticojejunostomy (HJ) for major BDI.

Patients and methods

This study was conducted at Al-Thawra Modern General Hospital in Sana'a, Yemen. In this retrospective cohort study, 54 consecutive patients with major iatrogenic BDI (Strasberg types D and E) were managed between 2014 and 2022 using a "physiology-first" protocol. Patients were stratified into four groups: immediate repair (<72 hours; G1, n = 22), early delayed (two to eight weeks; G2, n = 12), late delayed (≥3 months; G3, n = 12), and a critical care pathway for those presenting with sepsis or multi-organ failure (G4, n = 8). Definitive Roux-en-Y HJ was performed in 49 patients (90.7%), as five patients in G4 died during initial stabilization. The primary outcome was initial technical success. Long-term anastomotic patency survival was analyzed using Kaplan-Meier curves with log-rank testing, and independent predictors of repair failure were identified using multivariate logistic regression.

Results

The mean patient age was 52.4 ± 11.8 years, with a female predominance (66.7%, 36/54). Most injuries occurred during laparoscopic cholecystectomy (94.4%, 51/54), and 38.9% (21/54) were classified as high-grade (Strasberg E3-E5). The overall primary technical success rate was 77.8% (42/54). When stratified by protocol, success rates were 91.7% (11/12) for G3, 86.4% (19/22) for G1, 83.3% (10/12) for G2, and 37.5% (3/8) for G4. Major complications (Clavien-Dindo ≥III) occurred in 16.3% (8/49) of the surgical cohort. Long-term morbidity included anastomotic stricture in 20.4% (10/49), reoperation in 10.2% (5/49), and secondary biliary cirrhosis in 6.1% (3/49). Kaplan-Meier analysis at a median follow-up of 54 months (interquartile range (IQR) 38-58) demonstrated significantly inferior anastomotic patency survival for G2 compared to G3 (16.7% vs. 91.7% event-free; log-rank p < 0.001). Multivariate analysis identified sepsis/multi-organ failure at presentation (adjusted odds ratio (aOR) 10.00, 95% CI 1.26-79.4; p = 0.029) and high-grade injury (aOR 7.14, 95% CI 1.49-34.2; p = 0.014) as independent predictors of failure.

Conclusion

This study validates a "physiology-first" protocol for major iatrogenic BDI, demonstrating superior long-term results with late delayed repair (≥3 months). Crucially, the two-to-eight-week post-injury period was identified as a high-risk "danger zone" for reconstruction failure, challenging traditional early intervention paradigms in suboptimal biological conditions. These findings underscore the necessity of physiological optimization and specialized surgical timing to maximize anastomotic patency and survival, particularly in resource-constrained environments.

## Introduction

Laparoscopic cholecystectomy remains the gold standard treatment for symptomatic cholelithiasis and cholecystitis. Despite its advantages, iatrogenic bile duct injury (BDI) persists as a serious complication, with contemporary incidence rates reported between 0.3% and 0.7% [1,2]. A major BDI, defined as a Strasberg type D or E lesion, transforms a routine procedure into a life-altering event, leading to substantial long-term morbidity, repeated interventions, and a significant burden on healthcare systems [3].

The prevailing etiology of such injuries is often a cognitive-perceptual error involving the misidentification of anatomy, a risk that endures even among experienced surgeons, particularly in the setting of acute or chronic inflammation [4,5]. Once a major injury occurs, the optimal timing for surgical reconstruction remains a subject of intense debate, confounded by a lack of standardized chronological definitions in the literature [6,7]. While most experts categorize repair as immediate (<72 hours), early, or late, the specific cut-off points vary significantly between studies. For instance, some reports define "early" repair as occurring within 14 days, while others extend this window to six weeks, a period where local inflammation and tissue friability are often at their peak [7-9].

These discrepancies have produced conflicting data; while some high-volume centers advocate for intervention within 14 days to avoid prolonged biliary discontinuity, others demonstrate a sharp increase in anastomotic stricture rates, reaching as high as 40-60%, when reconstruction is attempted during the subacute phase [10,11]. Such inconsistency suggests that chronological time may be a poor surrogate for biological readiness. Factors implicated in the failure of the initial repair include concomitant vascular injury, ischemia from thermal damage, and reconstruction performed on an inflamed hilum [12-15]. Furthermore, reconstruction by a non-specialist and late referral to a hepatopancreatobiliary (HPB) center are recognized as critical modifiable factors contributing to poor outcomes [11,16].

This clinical dilemma is profoundly accentuated in resource-limited environments. In our regional context in Yemen, patients frequently experience delayed referrals, present with established biliary fistulas, or are in overt sepsis. These complex presentations are often mismatched with traditional repair paradigms established in high-volume global centers [16,17]. Consequently, robust data from such settings remain scarce, and existing studies are usually limited by small, heterogeneous cohorts and a lack of standardized reporting [13,18].

To address this gap, we developed and implemented a "physiology-first" management protocol. This approach prioritizes patient stabilization and the resolution of local inflammation over rigid adherence to early operative timelines. In line with recent international consensus calling for standardized reporting [19], the primary aim of this study was to evaluate the outcomes of this stratified protocol at a tertiary referral center in a resource-limited setting. We specifically sought to analyze the impact of repair timing on long-term patency and to identify the key predictors of surgical failure.

## Materials and methods

Study design and setting 

This retrospective, single-center cohort study was conducted at Al-Thawra Modern General Hospital, a tertiary hepatobiliary referral unit in Sana'a, Yemen. All consecutive adult patients (≥18 years) referred for surgical management of major iatrogenic BDI following cholecystectomy between January 2014 and December 2022 were included. The study received ethical approval from the Institutional Review Board of 21 September University (approval no.: 22-231A; date: January 22, 2024), with a waiver of informed consent granted due to the retrospective nature of data collection. The study followed the Strengthening the Reporting of Observational Studies in Epidemiology (STROBE) guidelines.

Patient selection

**Figure 1:**
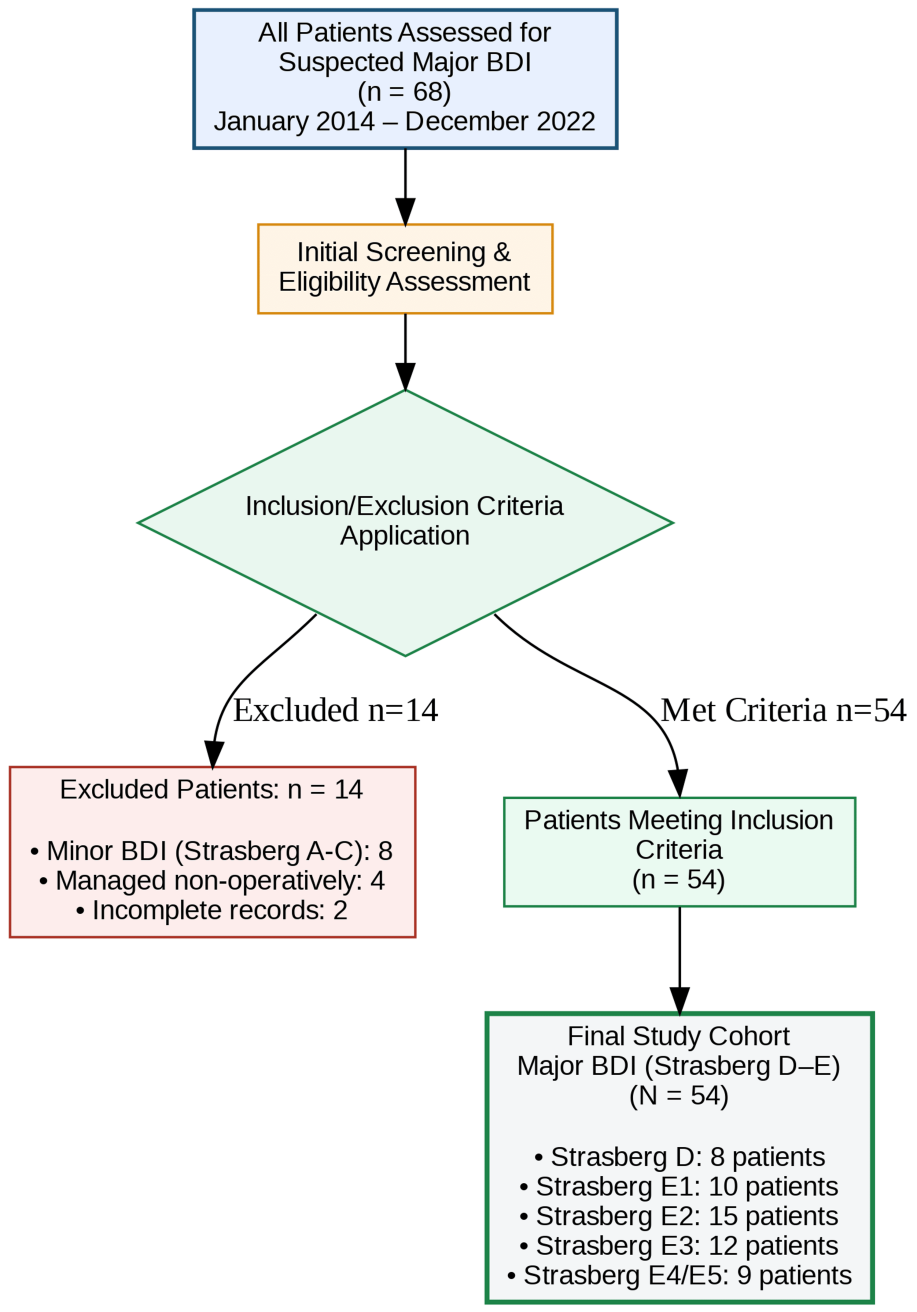
Study flow diagram (CONSORT-Style). Flowchart detailing the screening, inclusion, and exclusion of patients referred for major bile duct injury (BDI) management between 2014 and 2022. Of the initial patient cohort, 54 individuals met the inclusion criteria (Strasberg type D or E injury) and were stratified into the four management groups (G1-G4) of the "physiology-first" protocol for analysis. Image generated by the authors using Python (version 3.10) executed in a Google Colab environment, utilizing the Matplotlib and Seaborn libraries for data visualization and the Pandas library for data management.

Patients were included if they were adults (≥18 years) with a major BDI, defined as a Strasberg type D injury (lateral injury involving >50% of the duct circumference) or a type E injury (complete transection or stricture) [20]. Exclusion criteria were age <18 years (due to distinct pediatric biliary disease pathways), Strasberg types A-C injuries managed non-operatively, and referral for endoscopic management only. The patient selection process is summarized in Figure 1.

Data collection and variables

Data were extracted from archived medical records. Variables included patient demographics, details of the index cholecystectomy, time from injury to referral, clinical and laboratory status at presentation, Strasberg classification, operative details (including timing, technique, and findings), postoperative complications, and long-term outcomes. Long-term outcomes specifically encompassed anastomotic patency, episodes of cholangitis, and development of secondary biliary cirrhosis.

Initial assessment and triage protocol 

Upon referral, all patients underwent a standardized assessment focusing on physiological stabilization. This included clinical evaluation for sepsis or peritonitis, measurement of vital signs, laboratory markers (liver function tests, C-reactive protein, white blood cell count), and cross-sectional imaging (ultrasound or CT) to identify bilomas. Percutaneous transhepatic biliary drainage (PTBD) or endoscopic retrograde cholangiopancreatography (ERCP) with stent placement was performed for uncontrolled bile leaks, cholangitis, or proximal biliary obstruction. Preoperative biliary mapping was achieved via magnetic resonance cholangiopancreatography (MRCP) or direct cholangiography. Antibiotic therapy and nutritional support were provided to stabilize unstable patients before definitive repair.

Management protocol and patient stratification

**Figure 2:**
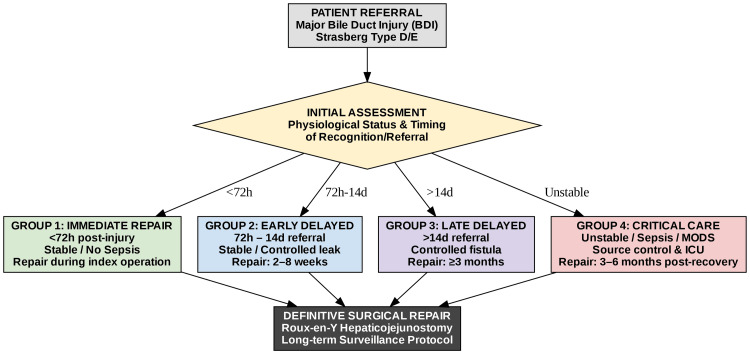
The "physiology-first" stratified management protocol. Schematic diagram illustrating the clinical triage and management pathways. Patient allocation into Immediate (<72 hours, G1), Early Delayed (two to eight weeks, G2), or Late Delayed (≥3 months, G3) repair pathways was determined by physiological stability and timing of referral. Patients presenting with sepsis or multi-organ failure (G4) entered a dedicated Critical Care Pathway prioritizing stabilization. Image generated by the authors using Python (version 3.10) executed in a Google Colab environment, utilizing the Matplotlib and Seaborn libraries for data visualization and the Pandas library for data management.

All definitive repairs were performed by a single hepatobiliary surgeon with 15 years of subspecialty experience. The timing of repair was determined based on existing literature [6], and patients were stratified into different pathways according to their clinical status and the timing of referral. Patients who were hemodynamically stable, with intraoperatively recognized major bile duct injuries, no signs of local inflammation or sepsis, and who underwent repair during the initial hospitalization, were classified as immediate repairs, performed within 72 hours (Group 1). Those who were clinically stable but referred more than 72 hours after injury, often with a controlled bile leak or localized inflammation, were scheduled for early delayed repair, within two to eight weeks (Group 2). Patients referred more than 14 days after injury, typically with an established biliary fistula, and who were stable, were categorized as late delayed, with elective reconstruction deferred for at least three months (Group 3). Patients presenting with sepsis, biliary peritonitis, or multi-organ dysfunction were managed through an intensive care pathway, focusing on stabilization, percutaneous drainage, and nutritional support. Definitive repair was only undertaken after complete physiological recovery, which usually occurred after a delay of 3 to 6 months (Group 4) (Figure 2).

Surgical technique 

**Figure 3:**
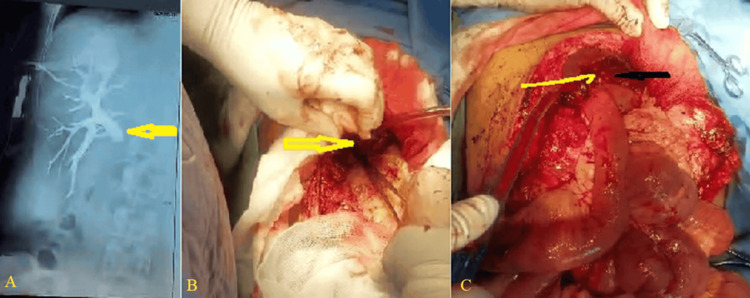
Surgical techniques for complex hilar reconstruction. (A) Preoperative cholangiogram revealing a complete transection of the common hepatic duct (Strasberg E4 injury) with contrast extravasation (yellow arrow). (B) Intraoperative view of the hilar plate following mobilization, showing the site of prior clip ligation (yellow arrow) and the ischemic common hepatic duct stump. (C) Completed Longmire procedure (left hepatic lobectomy), demonstrating the mucosa-to-mucosa, tension-free anastomosis between the left hepatic duct (black arrow) and the Roux-en-Y jejunal limb, achieving a wide-caliber bilioenteric conduit (yellow arrow). Image credit: created by the authors

The definitive procedure was a Roux-en-Y HJ with a 70-cm Roux limb. For complex hilar injuries (Strasberg E3-E5), the Hepp-Couinaud approach was employed, involving a longitudinal incision of the left hepatic duct to facilitate a wide, mucosa-to-mucosa anastomosis (Figure 3). In cases of severe inflammation, fibrosis, or densely adherent hilar plate, advanced techniques such as segment IV liver resection or Longmire mucosal graft HJ were utilized to access isolated segmental ducts or achieve a tension-free anastomosis. Anastomoses were fashioned with interrupted or continuous 4-0 or 5-0 polydioxanone (PDS) sutures (Figure 3).

Outcome measures 

The primary outcome was initial technical success, defined as the absence of anastomotic stricture and recurrent cholangitis (≥2 episodes per Tokyo Guidelines) at the end of follow-up [21]. Anastomotic patency survival was defined as freedom from a composite endpoint of anastomotic stricture, biliary reoperation, recurrent cholangitis (≥2 episodes per Tokyo Guidelines), or secondary biliary cirrhosis. Secondary outcomes included major morbidity (Clavien-Dindo Grade III or higher, e.g., those requiring surgical, endoscopic, or radiological intervention), treatment-related mortality, reoperation, and progression to secondary biliary cirrhosis.

Statistical analysis 

Data were analyzed using IBM SPSS Statistics for Windows, Version 28.0 (released 2021, IBM Corp., Armonk, NY). Continuous variables are presented as mean ± standard deviation or median with interquartile range (IQR), as appropriate. Categorical variables are expressed as frequencies and percentages. For subgroup analyses with n < 25, 95% confidence intervals for proportions were calculated using the Clopper-Pearson exact method. Group comparisons utilized Fisher’s exact test or χ² test for categorical variables, and ANOVA or Kruskal-Wallis tests for continuous variables.

Univariable logistic regression identified variables associated with repair failure. Variables with p < 0.10 and key clinical variables were included in multivariable logistic regression models, respecting an events-per-variable ratio >10:1 to prevent overfitting. Model discrimination was assessed via the C-statistic. Kaplan-Meier survival analysis was performed for anastomotic patency survival, with comparisons using the log-rank test. A two-sided p-value <0.05 was deemed statistically significant.

## Results

Patient cohort and clinical characteristics

A total of 54 patients underwent management for major iatrogenic BDIs between 2014 and 2022 at our tertiary hepatobiliary center. The mean age was 52.4 ± 11.8 years, with a female predominance (66.7%, 36/54). Laparoscopic cholecystectomy accounted for 94.4% (51/54) of index procedures. Strasberg classification identified 61.1% (33/54) low-grade injuries (types D, E1, E2) and 38.9% (21/54) high-grade injuries (E3-E5). Concomitant vascular injury occurred in 7.4% (4/54). At referral, 85.2% (46/54) were clinically stable, while 14.8% (8/54) presented with sepsis/multi-organ failure (MOF) (G4). Total bilirubin was highest in G4 (15.2 mg/dL, IQR 12.8-18.4; p < 0.001 vs. others) and serum albumin lowest (2.1 g/dL, IQR 1.8-2.4; p < 0.001), reflecting severe physiological derangement (Table 1).

**Table 1:** Baseline demographics and clinical presentation by management group. Notes: G1: immediate repair (<72 hours); G2: early delayed (two to eight weeks); G3: late delayed (≥3 months); G4: sepsis/multi-organ failure requiring stabilization. *p-values by ANOVA (continuous) or χ²/Fisher's exact (categorical); bold indicates significant group differences (Kruskal-Wallis for non-normal data). SD: standard deviation; IQR: interquartile range

Characteristic	G1 Immediate (n = 22)	G2 Early Delayed (n = 12)	G3 Late Delayed (n = 12)	G4 Critical Care (n = 8)	Total (N = 54)	p-value
Age (years), mean ± SD	51.2 ± 10.9	53.8 ± 12.4	52.1 ± 12.1	55.6 ± 11.2	52.4 ± 11.8	0.72
Female sex, n (%)	15 (68.2)	8 (66.7)	8 (66.7)	5 (62.5)	36 (66.7)	0.98
Laparoscopic cholecystectomy, n (%)	21 (95.5)	11 (91.7)	12 (100)	7 (87.5)	51 (94.4)	0.47
Strasberg classification						
Low-grade (D, E1-2), n (%)	14 (63.6)	7 (58.3)	8 (66.7)	4 (50.0)	33 (61.1)	0.81
High-grade (E3-E5), n (%)	8 (36.4)	5 (41.7)	4 (33.3)	4 (50.0)	21 (38.9)	
Concomitant vascular injury, n (%)	1 (4.5)	1 (8.3)	1 (8.3)	1 (12.5)	4 (7.4)	0.89
Total bilirubin (mg/dL), median (IQR)	6.8 (4.2-9.5)	8.2 (6.1-11.3)	7.5 (5.8-10.2)	15.2 (12.8-18.4)	8.9 (5.6-12.8)	<0.001
Serum albumin (g/dL), median (IQR)	3.2 (2.8-3.6)	2.9 (2.5-3.3)	3.1 (2.7-3.5)	2.1 (1.8-2.4)	2.9 (2.4-3.5)	<0.001

Management strategy and operative characteristics

Patients were managed via a "physiology-first" protocol and stratified into four clinical pathways. This included immediate repair within 72 hours (G1, n = 22), early delayed repair between two and eight weeks (G2, n = 12), and late delayed repair at or after three months (G3, n = 12) for stable patients. Critically ill patients presenting with sepsis or MOF (G4, n = 8) required intensive care stabilization, during which five patients died preoperatively. Consequently, 49 patients (90.7%) proceeded to definitive Roux-en-Y HJ. Advanced surgical techniques, such as segment IV resection (n = 4) and Longmire procedures (n = 5), were successfully utilized in 42.9% (9/21) of the high-grade injury subset.

Primary outcomes

The overall primary technical success rate was 77.8% (42/54). When stratified by management group, success rates were 91.7% (11/12) for G3, 86.4% (19/22) for G1, 83.3% (10/12) for G2, and 37.5% (3/8) for G4. Inter-group comparisons demonstrated a significant overall difference in success (p < 0.001, Table 2).

**Table 2:** Primary outcomes stratified by management protocol. Notes: †Not applicable (5 G4 patients died during stabilization). Statistical comparisons: Overall χ² p < 0.001. Pairwise (Fisher's exact): G1 vs. G2 p = 1.00, G1 vs. G3 p = 0.681, G1 vs. G4 p < 0.001, G2 vs. G3 p = 1.000, G2 vs. G4 p = 0.02, G3 vs. G4 p < 0.001. MOF: multi-organ failure

Management group	n	Median time to surgery	Primary success, n (%)	Mortality, n (%)
G1: Immediate (<72h)	22	1 day	19 (86.4)	0 (0.0)
G2: Early Delayed (2-8w)	12	38 days	10 (83.3)	0 (0.0)
G3: Late Delayed (≥3mo)	12	102 days	11 (91.7)	0 (0.0)
G4: Critical Care	8	N/A†	3 (37.5)	5 (62.5)
Total	54	-	42 (77.8)	5 (9.3)

Postoperative complications and long-term morbidity

Among the 49 patients who underwent biliary reconstruction, the 90-day morbidity rate was 26.5% (13/49). Major complications, defined as Clavien-Dindo grade III or higher, occurred in 16.3% (8/49), comprising grade IIIb (n = 6) and grade IVa (n = 2) events, with no recorded grade V mortality post-repair (Table 3).

**Table 3:** Postoperative complications (surgical cohort, N = 49). Note: 95% CI calculated using Wilson score method HJ: hepaticojejunostomy

Outcome	n (%)	95% CI
Early complications (≤90 days)		
Bile leak	4 (8.2)	2.3-19.6
Intra-abdominal abscess	5 (10.2)	3.4-22.2
Major morbidity (Clavien-Dindo ≥III)	8 (16.3)	7.3-29.7
Grade IIIb	6 (12.2)	4.5-25.1
Grade IVa	2 (4.1)	0.5-14.0
Grade V	0 (0.0)	-
Late complications		
Anastomotic stricture	10 (20.4)	10.2-34.3
Median time to stricture, months (IQR)	14.5 (8.0-22.0)	-
Reoperation (redo-HJ)	5 (10.2)	3.4-22.2
Secondary biliary cirrhosis	3 (6.1)	1.3-16.9

Anastomotic stricture affected 20.4% (10/49) of the cohort at a median follow-up of 54 months (IQR: 42.0-68.0). These strictures occurred significantly more often in G2 (6/12, 50.0%) compared to G3 (1/12, 8.3%; p = 0.016). Redo HJ was required in 10.2% (5/49) of cases, achieving an 80% success rate, while secondary biliary cirrhosis developed in 6.1% (3/49) (Table 4).

**Table 4:** Long-term outcomes by management group. Notes: G2 versus G3 strictures (Fisher's exact p = 0.016). Events are non-mutually exclusive. †G4 surgical survivors only (3/8 total G4 patients).

Group	n	Total events n (%)	Stricture n (%)	Cholangitis n (%)	Redo HJ n (%)	Cirrhosis n (%)
G1	22	11 (50.0)	2 (9.1)	2 (9.1)	3 (13.6)	0 (0.0)
G2	12	11 (91.7)	6 (50.0)	3 (25.0)	2 (16.7)	1 (8.3)
G3	12	2 (16.7)	1 (8.3)	1 (8.3)	0 (0.0)	0 (0.0)
G4†	3	3 (100.0)	1 (33.3)	1 (33.3)	2 (66.7)	2 (66.7)
Total	49	27 (55.1)	10 (20.4)	7 (14.3)	7 (14.3)	3 (6.1)

Kaplan-Meier analysis

**Figure 4:**
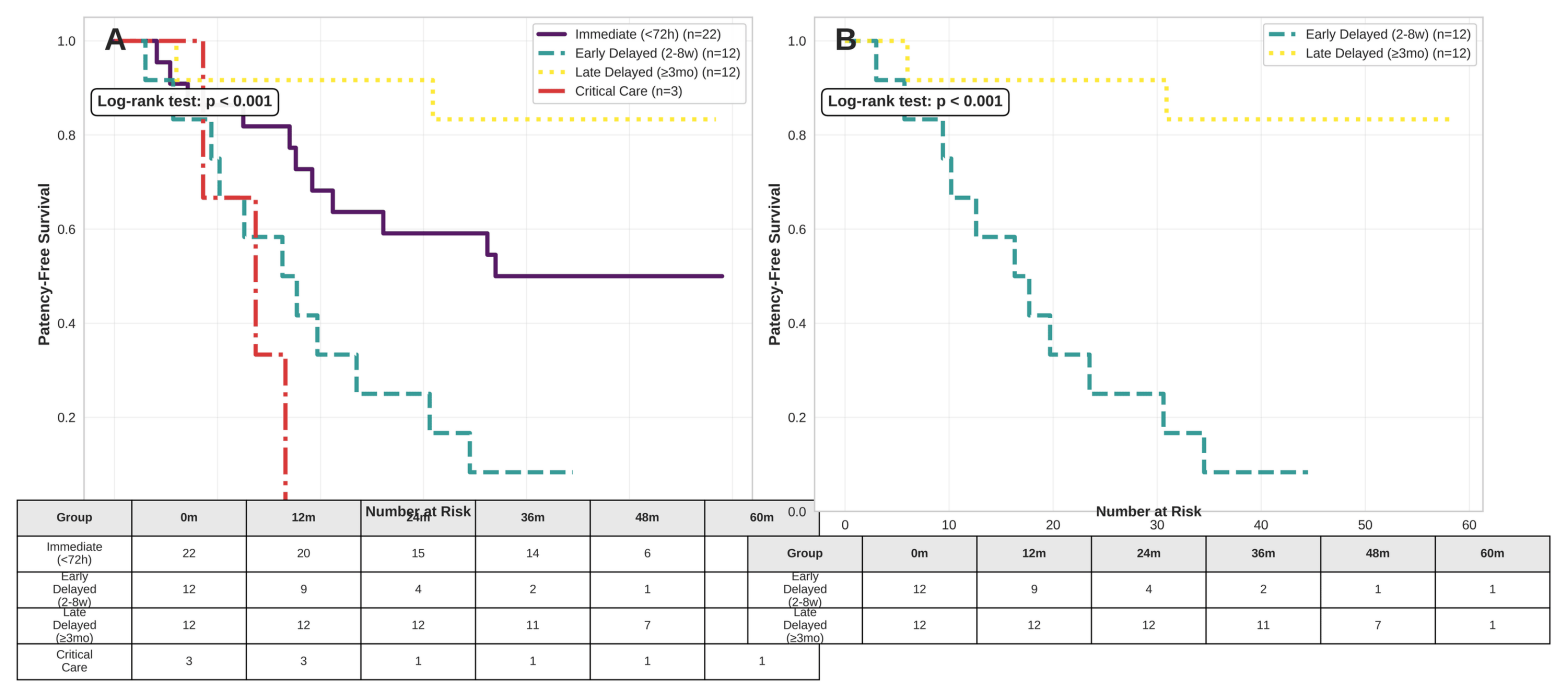
Kaplan-Meier analysis of patency-free survival. (A) Overall protocol comparison: survival curves representing freedom from the composite endpoint (anastomotic stricture, reoperation, secondary biliary cirrhosis, or recurrent cholangitis) for all management groups (log-rank p < 0.001). Event counts were as follows: G1, n = 11; G2, n = 11; G3, n = 2; G4, n = 3. (B) Early vs. late delayed repair: head-to-head comparison confirming the superiority of late delayed (G3) over early delayed (G2) repair. At 54 months, 91.7% (11/12) of G3 patients remained event-free versus 16.7% (2/12) in G2 (hazard ratio 8.2, 95% CI 1.8–37.4; log-rank p < 0.001), identifying the two-eight-week interval as a high-risk period for failure. Image generated by the authors using Python (version 3.10) executed in a Google Colab environment, utilizing the Matplotlib and Seaborn libraries for data visualization and the Pandas library for data management.

Kaplan-Meier analysis of anastomotic patency survival, defined as freedom from the composite endpoint of anastomotic stricture, reoperation, secondary biliary cirrhosis, or recurrent cholangitis, demonstrated significant divergence between management groups (log-rank p < 0.001; Figure 4A). A direct comparison confirmed the clinical superiority of late delayed repair (Group 3) over early delayed repair (Group 2), with 91.7% versus 16.7% of patients remaining event-free at 54 months (hazard ratio (HR) 8.2, 95% CI 1.8-37.4; log-rank p < 0.001; Figure 4B).

Predictors of primary repair failure

Multivariate logistic regression identified sepsis or MOF at presentation as the strongest independent predictor of primary repair failure (adjusted odds ratio (aOR) 10.00, 95% CI 1.26-79.4; p = 0.029), followed by high-grade injury (Strasberg E3-E5) (aOR 7.14, 95% CI 1.49-34.2; p = 0.014) (Table 5). Concomitant vascular injury also showed a strong univariate association (OR 7.50, 95% CI 1.42-39.6; p = 0.008). While the timing of repair (G1 or G2 vs. G3 reference) did not achieve independent statistical significance in the regression model (aOR 1.56-2.38; p = 0.37), likely due to limited statistical power, the Kaplan-Meier analysis unequivocally demonstrated the clinical advantage of late delayed repair (G3). The model showed excellent discriminative ability with a c-statistic of 0.84 (Hosmer-Lemeshow p = 0.72).

**Table 5:** Predictors of primary repair failure (univariate and multivariate analysis). **p < 0.05 (statistically significant). Model performance: c-statistic = 0.84; Hosmer-Lemeshow goodness-of-fit p = 0.72 G3 (late delayed, ≥3 months) as reference group for timing variables. Vascular injury not entered in multivariate model due to low event numbers (n = 4). OR: odds ratio; aOR: adjusted odds ratio; CI: confidence interval; MOF: multi-organ failure

Variable	Univariate OR (95% CI)	p-value	Multivariate aOR (95% CI)	p-value
Sepsis/MOF at presentation	9.33 (2.35-37.1)	0.001*	10.00 (1.26-79.4)	0.029*
High-grade injury (E3-E5)	5.42 (1.64-17.9)	0.005*	7.14 (1.49-34.2)	0.014*
Vascular injury	7.50 (1.42-39.6)	0.008*	-	-
Bilirubin >10 mg/dL	1.78 (0.56-5.65)	0.329	1.89 (0.46-7.81)	0.378
Timing: G1 vs. G3	1.58 (0.33-7.57)	0.567	1.56 (0.25-9.70)	0.620
Timing: G2 vs. G3	2.27 (0.45-11.5)	0.322	2.38 (0.34-16.5)	0.370
Age >60 years	1.25 (0.38-4.11)	0.718	1.27 (0.31-5.17)	0.742
Female sex	0.67 (0.23-1.97)	0.467	0.76 (0.19-3.02)	0.694

## Discussion

Main findings

This study demonstrates that late delayed reconstruction (≥3 months) achieved superior technical success compared to early delayed repairs in managing 54 consecutive major BDIs at Yemen's sole tertiary center. The overall success rate was 77.8%, with sepsis at presentation and high-grade injury (Strasberg E3-E5) independently predicting failure. These findings support the concept that physiology-optimized timing is superior to rigid early repair protocols, particularly challenging the established two-eight-week "danger zone," during which inflammation contributes to higher stricture rates and an increased risk of composite adverse events.

Etiology of BDI

Recent literature indicates that iatrogenic BDI rates in laparoscopic practice remain between 0.3% and 0.7%, a significant increase from the 0.1% to 0.2% historically observed in open procedures [22,23]. This persistent risk is reflected in our cohort, where 94.4% of injuries occurred during laparoscopic cholecystectomy. The increase in BDI rates during the laparoscopic era may be attributed to a combination of the "misperception" phenomenon and variations in surgical training [24]. While some suggest a lack of experience during residency contributes to these rates, our data and existing literature indicate that cognitive-perceptual errors remain a threat even among surgeons beyond their learning curve [4].

Current evidence suggests that the majority of major injuries stem from cognitive-perceptual errors, specifically the misidentification of biliary anatomy, rather than technical inexperience [24]. This reality underscores the vital role of preventive strategies, specifically cognitive aids such as the consistent attainment of the "Critical View of Safety" (CVS) and the use of intraoperative cholangiography, as standardized safeguards to mitigate the risk of injury [25].

Treatment modality

The primary objective in the surgical management of BDI is to restore biliary-enteric continuity, thereby achieving durable, symptom-free outcomes and minimizing the need for subsequent intervention. At this institution, Roux-en-Y HJ is the procedure of choice for the repair of major BDIs. This approach is consistent with the findings of Ray et al. and aligns with the broader surgical literature, which identifies Roux-en-Y HJ as the predominant and preferred reconstructive technique for BDI, supported by evidence for its superior long-term anastomotic patency and clinical results [1,26,27].

In our study, repair involving liver resection, either wedge resection of segment IVb or formal left lobectomy (Longmire procedure), was required in nine patients (16.7% of the cohort). This subset universally involved high-grade injuries (Strasberg E3-E5), where inflammation, fibrosis, or the anatomical retraction of ducts precluded a standard hilar dissection. The principal rationale for resection aligns with established principles of complex biliary reconstruction: to achieve adequate exposure of healthy, well-vascularized proximal ducts and to create a tension-free, mucosa-to-mucosa HJ [15,28,29]. While this approach successfully facilitated reconstruction in all cases, the necessity for parenchymal resection itself served as a marker of injury severity, which was subsequently associated with a greater propensity for long-term complications, including anastomotic stricture and progression to cirrhosis [29]. Our experience reinforces that in complex proximal BDIs, a surgeon's preparedness to employ advanced techniques, including limited hepatic resection, is paramount to achieving definitive repair, albeit with the recognition that these injuries portend a more challenging postoperative course.

Timing of surgical repair

The optimal timing for surgical reconstruction of major BDI is nuanced, informed by a confluence of patient physiology, local hilar conditions, and institutional expertise. Our study, employing a stratified "physiology-first" protocol, adds a critical dimension to this debate. We found that while initial technical success was comparable between Immediate (<72 hours, 86.4%) and Late Delayed (≥3 months, 91.7%) repairs, long-term anastomotic patency survival was significantly superior in the Late Delayed group compared to those repaired in the Early Delayed (2-8 weeks) interval (91.7% vs. 16.7%, log-rank p < 0.001).

This stark contrast is consistent with the "danger zone" hypothesis advanced by Manivasagam et al. and the meta-analysis by Schreuder et al., which recommend avoiding the two-to-six week period due to significantly higher morbidity and stricture rates [7,30]. Our Kaplan-Meier analysis strongly corroborates this, suggesting that the subacute inflammatory phase is biologically unfavorable for durable healing. Our findings specifically align with the meta-analysis by Schreuder et al., which advocates for avoiding the subacute phase; however, our 91.7% success rate for late delayed repair (G3) mirrors the upper range of patency rates reported in pooled international data, validating the "physiology-first" approach even in resource-limited contexts [30]. This finding, however, exists within a spectrum of reported outcomes. Some high-volume centers, such as Giuliante et al., report excellent success with Hepp-Couinaud hepatico-jejunostomy regardless of timing in stable patients with favorable anatomy (dilated ducts, no leak), prioritizing surgical technique [8]. Conversely, the large E-AHPBA study found that while anastomotic patency was unaffected by timing, early and intermediate repairs were associated with a fourfold higher biliary-related mortality compared to late repair [31].

This underscores a vital distinction: technical feasibility is not equivalent to optimal patient safety. Therefore, the accumulating evidence, including our own, supports a paradigm where delayed repair is the preferred strategy for stable patients, as it allows inflammation to resolve, thereby optimizing both safety and long-term patency. The decision for immediate repair should be reserved for specific, controlled scenarios or when mandated by a patient's deteriorating clinical course, with the understanding that it may carry a different risk profile for long-term stricture. However, the interpretation of our results, particularly concerning the optimal timing of repair, must be considered within the context of a persistent challenge in this field: the absence of standardized definitions, as detailed in a recent systematic review where "immediate repair" ranged from <24 hours to six weeks post-injury [6].

Postoperative complications

This study underscores the necessity of long-term surveillance, as complications following Roux-en-Y HJ can arise years after surgery. Despite a median follow-up of 54 months, the rate of anastomotic stricture in our cohort was 20.4%, which is notably higher than the 10-19% reported in prior literature [28,29,32,33]. ERCP and PTBD served as the primary modalities for biliary decompression in our series, although emerging techniques such as endoscopic ultrasound (EUS)-guided drainage offer a valuable alternative for source control in equipped centers [34]. While these findings reflect the outcomes of a single-center, single-operator experience, they highlight the technical challenges inherent in managing high-grade injuries. These strictures were initially managed with endoscopic stenting (n = 4) or percutaneous drainage (n = 6); however, eight patients developed recurrent cholangitis, five of whom underwent redo HJ (successful in four cases, 80%). Ultimately, three patients progressed to secondary biliary cirrhosis, requiring evaluation for orthotopic liver transplantation. This pattern of delayed and complex sequelae strongly aligns with findings by Schreuder et al. and Sulpice et al., reinforcing the critical need for vigilant, lifelong follow-up to optimize long-term outcomes after biliary reconstruction [32,35].

Factors associated with failure

Our findings contribute to the evolving understanding of risk factors predictive of adverse outcomes following surgical repair of major BDIs. The literature consistently identifies postoperative bile leak, prior unsuccessful repair attempts, and sepsis at the time of reconstruction as factors associated with increased anastomotic stricture and morbidity [8,13,36-38]. In the context of BDI, we defined morbidity using the Clavien-Dindo classification, specifically focusing on grade III or higher complications, such as enteric fistulas and recurrent cholangitis, that directly impact repair longevity. Our multivariate analysis corroborates and refines this profile, identifying sepsis or MOF at presentation (aOR 10.00, p = 0.029) and high-grade injury (Strasberg E3-E5) (aOR 7.14, p = 0.014) as the strongest independent predictors of primary repair failure. The potent association of sepsis aligns precisely with the work of Sulpice et al., who identified it as a critical determinant of both major morbidity and long-term stricture [32]. In contrast to Hajjar et al. [29], postoperative bile leak was not an independent predictor in our model. This discrepancy may stem from differences in perioperative management or, more likely, indicates that the profound prognostic influence of presenting physiology and injury anatomy in our cohort attenuated the independent statistical contribution of other factors.

These factors must guide risk stratification and emphasize the non-negotiable imperative for complete physiological optimization, particularly in septic patients, before undertaking definitive reconstruction, as evidenced by the outcomes in our critically ill cohort.

Clinical implications and management in resource-limited settings

These findings underscore several fundamental clinical imperatives. First, they argue compellingly for the prompt referral of patients with major BDI to specialized hepatobiliary centers, a practice consistently associated with improved outcomes [6,19]. This is particularly relevant given the rising rates of BDI, while some suggest that a lack of residency-based experience contributes to these trends, our protocol emphasizes that specialized "physiology-first" management can mitigate these risks even in high-complexity cases. Second, they reinforce that management must be guided by continuous physiological assessment rather than rigid timelines. Our stratified protocol, which identified the 2-8 week period as a high-risk interval for long-term failure, provides a practical framework for balancing timely intervention with the necessity for a safe operative environment.

In regions where immediate access to specialized hepatopancreatobiliary (HPB) care is limited, initial management must focus on stabilization. Adhering to a "drain now, fix later" principle is essential. This involves percutaneous drainage of bilomas, administration of targeted antibiotics for source control, and provision of nutritional support to address the systemic inflammatory response. Such measures mitigate sepsis, reduce localized inflammation, and create a more favorable scenario for eventual definitive reconstruction. Crucially, complex biliary reconstruction should not be attempted without requisite expertise, as non-specialist repair is a known predictor of failure [19,39]. This staged approach prioritizes physiological stabilization and safe referral over premature surgical intervention, thereby actively avoiding the high-failure interval identified in our analysis.

Study limitations

This study carries several limitations inherent to its retrospective, single-center design performed by a single surgeon. While this ensures technical consistency, it introduces potential performer bias and may limit the generalizability of results to centers with different levels of specialized expertise. As treatment allocation was determined by a standardized institutional protocol rather than randomization, the potential for selection bias must be acknowledged. Although the cohort size is substantial for a major BDI series, the total sample size (N = 54) remains relatively small, which may limit the statistical power to detect rare predictors of repair failure. The median follow-up of 54 months, while robust, may not capture very late sequelae emerging beyond a decade. Furthermore, this study focused specifically on major injuries (Strasberg types D and E); consequently, no long-term follow-up was conducted for type A and B injuries, representing a gap in the longitudinal tracking of the full spectrum of BDI subtypes. Outcomes are intrinsically linked to the expertise of a high-volume tertiary center and may not be directly generalizable to lower-resource settings. In addition, while we addressed cognitive-perceptual errors, we did not explicitly analyze the correlation between current surgical residency training curves and the reported increase in BDI rates. Additional unmeasured confounders, such as specific comorbidities, detailed nutritional status, and precise ductal diameters, likely influenced results. Notably, a formal comparison between our surgical outcomes and emerging minimally invasive alternatives, such as EUS-guided drainage, was not performed as this modality was not available during the study period. Finally, the lack of standardized quality-of-life metrics limits the assessment of functional recovery. These limitations highlight specific targets for future research. Prospective, multicenter studies are needed to validate the "physiology-first" protocol and the optimal duration of delayed repair, formally compare surgical repair against other interventions, and evaluate adapted strategies for diverse healthcare environments.

## Conclusions

Our findings demonstrate that effective management of major bile duct injury demands a stratified, "physiology-first protocol." Definitive reconstruction should be deferred until full physiological stabilization occurs, intentionally avoiding the high-risk subacute period of two to eight weeks post-injury. This strategy achieved a 91.7% success rate with late delayed repair. Sepsis at presentation and high-grade injury emerged as the dominant outcome predictors, confirming that physiological and anatomical factors outweigh rigid timelines in surgical decision-making. Although derived from single-center experience, these results support a paradigm shift from urgent repairs in suboptimal conditions toward proactive triage, delayed reconstruction in expert hands, and routine early referral to specialized hepatobiliary centers. Ultimately, this physiology-first approach optimizes long-term hepatobiliary outcomes and minimizes morbidity from these devastating injuries.
